# Multimodality imaging of ischaemia with non-obstructive coronary artery disease in a patient with coronary artery ectasia: a case report

**DOI:** 10.1093/ehjcr/ytaf246

**Published:** 2025-05-16

**Authors:** Masatoki Nakaza, Yukihiro Watanabe, Keishi Suzuki, Akira Shibata, Masashi Ogawa, Tetsuro Sekine

**Affiliations:** Department of Radiology, Nippon Medical School, 1-1-5 Sendagi, Bunkyo-ku, Tokyo 113-8603, Japan; Department of Cardiovascular Medicine, Nippon Medical School, 1-1-5 Sendagi, Bunkyo-ku, Tokyo 113-8603, Japan; Department of Cardiovascular Medicine, Nippon Medical School, 1-1-5 Sendagi, Bunkyo-ku, Tokyo 113-8603, Japan; Department of Cardiovascular Medicine, Nippon Medical School, 1-1-5 Sendagi, Bunkyo-ku, Tokyo 113-8603, Japan; Department of Radiology, Nippon Medical School, 1-1-5 Sendagi, Bunkyo-ku, Tokyo 113-8603, Japan; Department of Radiology, Nippon Medical School Musashi Kosugi Hospital, 1-383 Kosugimachi, Nakahara-ku, Kawasaki-shi, Kanagawa 211-8533, Japan

**Keywords:** Coronary artery ectasia, Coronary artery disease, Coronary computed tomography angiography, Cardiac nuclear imaging, Four-dimensional flow coronary magnetic resonance imaging

A 76-year-old woman was hospitalized for heart failure. Echocardiography demonstrated a preserved left ventricular ejection fraction of 62%, but wall motion abnormalities were detected in the inferior wall. Coronary computed tomography angiography showed coronary artery ectasia (CAE) in three arteries without significant stenosis (*Figure 1*A and *B*). 13N-ammonia positron emission tomography imaging revealed rest perfusion defect with larger stress perfusion defect in the inferior and inferoposterior wall, which indicates myocardial infarction with concomitant myocardial ischaemia (*Figure 1C*: polar map stress and *D*: polar map rest). The myocardial blood flow reserve in each of the three arterial territories decreased, particularly in the right coronary artery (RCA) territory at 1.43. Four-dimensional flow coronary magnetic resonance imaging revealed slow and turbulent flow in the proximal RCA [*Figure 1E* and *F*: forward flow (white arrow) and backward flow (white arrowhead)]. Coronary flow volume (time-averaged flow volume) in the RCA was 0.15 mL/s in one cardiac cycle (*Figure 1G*: coronary flow velocity waveform).^1^ A similar, albeit less pronounced, flow pattern was observed in the left main trunk (measured value, 0.38 mL/s).

**Figure 1:**
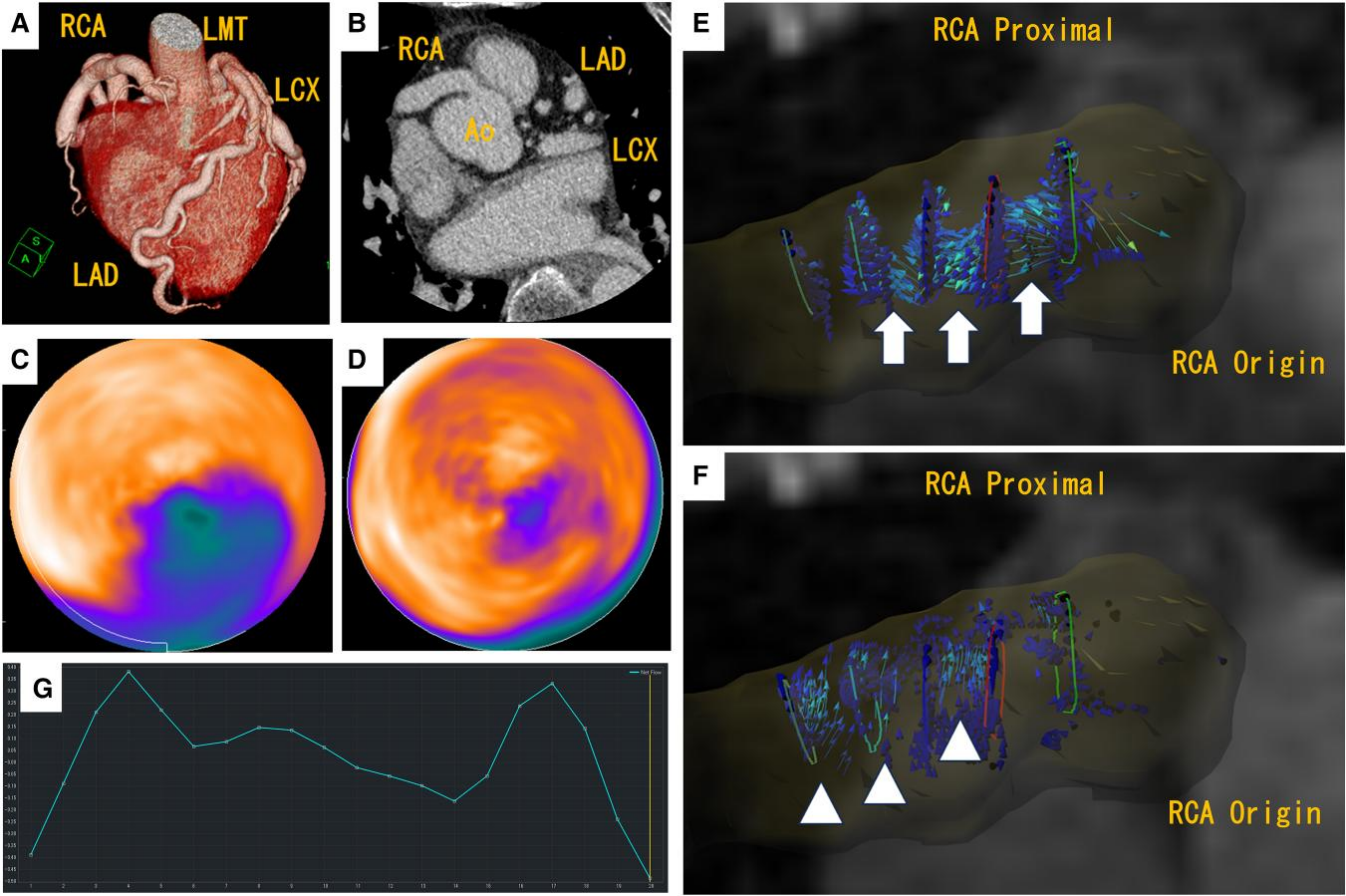
(*A*) and (*B*) Coronary computed tomography angiography. (*A*) Volume rendering imaging and (*B*) axial imaging. Coronary computed tomography angiography revealed coronary artery ectasia in all three coronary arteries without significant stenosis. (*C*) and (*D*) Polar maps from 13N-ammonia positron emission tomography. (*C*) Stress imaging and (*D*) rest imaging. The positron emission tomography images revealed a rest perfusion defect with a larger stress perfusion defect in the inferior and inferoposterior walls, indicating myocardial infarction with concomitant myocardial ischaemia. (*E*) and (*F*) Four-dimensional flow coronary magnetic resonance imaging. These panels demonstrate slow and turbulent flow in the proximal right coronary artery. Forward flow is indicated by a white arrow and backward flow by a white arrowhead. (*G*) Coronary flow velocity waveform. The waveform lacks the typical biphasic pattern with a predominant diastolic phase. Blood flow velocity is generally reduced, the waveform is irregular, and the peak is poorly defined. These findings suggest the presence of abnormal flow, such as slow or turbulent flow.

Coronary artery ectasia is defined as a diffuse or focal dilatation of the epicardial coronary artery and has gained increasing recognition for its association with non-obstructive coronary artery disease;^2^ however, its underlying pathophysiology remains poorly understood. This case demonstrated pronounced epicardial coronary flow disturbances in the RCA accompanied by myocardial ischaemia and coronary microvascular dysfunction in the perfusion territory. These findings highlight the key role of the combination of epicardial flow disturbances and microvascular dysfunction in the pathogenesis of myocardial ischaemia in patients with CAE.^3^

We used a SIGNA Architect 3.0T MRI system (GE Healthcare) and GTFlow software (GyroTools) for 4D flow MRI analysis. The detailed scan parameters were as follows: repetition time/echo time = 4.0/2.7 ms; flip angle = 13°; voxel size = 1.3 × 1.3 × 2.0 mm³; temporal resolution = 83 ms; velocity encoding = 50 cm/s; 20 cardiac phases; HyperKat acceleration (acceleration factor = 8); and navigator-based respiratory gating with a respiratory compensation acceptance ratio of 25%.

**Consent:** Written informed consent was obtained from the patient for the publication of this case report and the accompanying images.

**Funding**: None declared.

## Data Availability

All relevant data are included in the article.
